# Dietary supplementation with full-fat *Hermetia illucens* larvae and multi-probiotics, as a substitute for antibiotics, improves the growth performance, gut health, and antioxidative capacity of weaned pigs

**DOI:** 10.1186/s12917-022-03550-8

**Published:** 2023-01-11

**Authors:** Pheeraphong Phaengphairee, Waewaree Boontiam, Alexandra Wealleans, Jinsu Hong, Yoo Yong Kim

**Affiliations:** 1 Division of Animal Science, Faculty of Agriculture, Khon Kaen University, Khon Kaen, 40002 Thailand; 2 Kemin Europa N.V., Animal Nutrition and Health EMENA, Toekomstlaan 42, 2200 Herentals, Belgium; 3 Department of Animal Science, South Dakota State University, Brookings, SD 57007 USA; 4 School of Agricultural Biotechnology, and Research Institute of Agriculture and Life Sciences, Seoul National University, Gangnam-ru, Seoul, 135-754 South Korea

**Keywords:** Black soldier fly, *Bacillus* probiotics, Antibiotic-free, Piglets, Gut health, Oxidative stress

## Abstract

**Background:**

Dietary supplementation of full-fat black soldier fly larvae (BSFL full-fat meal; alone or in combination with multi-probiotics) was tested as an alternative to dietary antibiotics in weaning piglets. We also tested the effects of these diets on growth performance, nutrient digestibility coefficients, immune status, oxidative stress, intestinal histomorphology, and rectal microbial modulations in weaned pigs. A total of 80 piglets [(Landrace × Large White) × Duroc] of both sexes (a ratio of gilts and barrows; 1:1), were randomly allotted to four diet groups: positive control (PC) diet supplemented with 0.02% amoxicillin; negative control (NC) diet without supplement addition; BSFL12 diet (NC + 12% BSFL full-fat meal); and BSFL + Pro diet (BSFL full-fat meal + 0.1% multi-probiotics, including *Bacillus subtilis*, *B. licheniformis*, and *Saccharomyces cerevisiae*). All groups had five replicates, with four piglets per replicate.

**Results:**

Dietary BSFL + Pro improved the overall average daily gain (*P* = 0.013), and gain-to-feed ratio (*P* = 0.032). The BSFL12 and BSFL + Pro diets improved nutrient digestibility and increased the serum levels of immunoglobulin A and glutathione peroxidase, while reducing the levels of pro-inflammatory cytokines. The spleen weight was higher and caecal pH was lower in pigs fed the BSFL + Pro diet than in those fed the NC diet (*P* = 0.011 and *P* = 0.021, respectively). Pigs fed the BSFL diets had longer duodenal villi, a higher villus height-to-crypt depth ratio (*P* = 0.004), and shorter crypt depth (*P* = 0.017) than those fed NC. The BSFL + Pro diet also increased faecal *Lactobacillu*s spp. count (*P* = 0.008) and reduced *Escherichia coli* (*P* = 0.021) counts compared with that seen with PC and NC diets, respectively.

**Conclusions:**

Dietary supplementation with BSFL or BSFL + multi-probiotics can improve the growth performance and intestinal health of pigs and may be an effective strategy to replace antibiotics for weaned pigs.

## Background

For several years, amoxicillin has been widely used to control post-weaning diarrhoea in piglets. However, the excessive use of antimicrobials in food animal production has led to concerns regarding the spread of multidrug-resistant pathogens. Since 28 January 2022, the use of amoxicillin in the food animal production industry has been regulated by the European Medicines Agency [1]. To curb the escalating problem of antibiotic resistance, the bioactive properties of black soldier fly larva full-fat meal (BSFL) and multi-strain probiotics (i.e., *Bacillus subtilis*, *B. licheniformis*, and *Saccharomyces cerevisiae*) have been suggested as effective alternatives to in-feed antibiotics. The BSFL is an attractive protein source for monogastric animals, especially because of its high crude protein content (40.11 ± 7.1%), ether extract (32.54 ± 11.2%), essential amino acid content (0.7 ± 0.4% methionine, 2.3 ± 0.9% lysine, and 1.8 ± 1.2% arginine), and lauric acid content (64 ± 7.8%) [2–5]. BSFL-derived antimicrobial peptides (AMPs) and chitin also inhibit the proliferation of harmful bacteria and activate immunity [6, 7]. Previous studies have demonstrated that the BSFL full-fat meal can be used in dietary formulations at a concentration of 8% in weanling pigs [8] and 18.5% in growing pigs [9], without any detrimental effects on growth performance. In addition, multi-strain probiotics are well-established to have better antibacterial, anti-inflammatory, and antioxidant properties than single-strain probiotics [10, 11]. Recently, a multi-strain *Bacillus subtilis*-based probiotic has been shown to improve the growth performance of pigs and the nutrient digestibility of essential amino acids [12]. Moreover, the inclusion of up to 0.3% probiotic mixture improves the growth performance of weaning pigs, inhibits the growth of harmful microbes, and reduces faecal NH_3_ emissions [13].

To our knowledge, few studies have examined how BSFL and multi-strain probiotics (alone or in combination), when used as substitutes for amoxicillin, affect growth performance, nutrient digestibility coefficients, immune status, oxidative stress, intestinal histomorphology, and rectal microbial modulation in weaned pigs. We hypothesised that when used in combination, BSFL full-fat meal and multi-strain probiotics were more likely to enhance the growth performance and gut health of weaned pigs than in-feed antibiotics. This would help mitigate the problem of antimicrobial resistance due to the escalating use of amoxicillin.

## Methods

This study was reported in accordance with ARRIVE guidelines and was conducted in strict accordance with the guidelines of the National Research Council of Thailand. Protocols were approved by the Institutional Animal Care and Use committee of Khon Kaen University (Khon Kaen, Thailand; approval number 125/64 on 18 November, 2021).

### BSFL and probiotics

The BSFL (*Hermetia illucens*) were obtained locally (Ban Dangnoi, Khon Kaen, Thailand), reared under constant conditions (27 ± 1 °C, 65% relative humidity, and L12:D12 photoperiod) and fed broiler feed substrate without any feed additive, coccidiostats, or antibiotic growth promoters (expect for 29 mg of CuSO_4_ and 108 mg of ZnSO_4_). Larvae were harvested on day 13 of larval development and killed by freezing at − 20 °C for 6 h. Next, the samples were dried at 65 °C for 72 h, and their nutrient composition was analysed before they were added to the dietary formulation. The nitrogen-to-protein conversion factor (*k*_*p*_) for BSFL full-fat meal is 5.33 [14]. This was subsequently used to calculate crude protein percentage as 5.33 x the nitrogen (N) content. The main components of BSFL are crude protein (CP, 35.88%), ether extract (EE, 30.58), lysine (2.31%), total essential amino acids (15.81%), non-essential amino acids (18.59%), lauric acid (9.17%), total saturated fatty acids (21.09%), unsaturated fatty acids (11.62%), and chitin (4.26%) (Table 1). The multi-probiotics included *Bacillus subtilis* (1 × 10^11^ cfu/kg), *Bacillus licheniformis* (1 × 10^9^ cfu/kg), and *Saccharomyces cerevisiae* (1 × 10^9^ cfu/kg).

**Table 1:** Analysed values of BSF larvae (*Hermetia illucens,* dry matter basis)

Item	Amount (g/100 g)	Item	Amount (g/100 g)
Dry matter	92.36	Saturated fatty acids (SFA)	
Total ash	11.74	Butyric acid (C4:0)	ND
Crude protein	35.88	Caproic acid (C6:0)	ND
Ether extract	30.58	Caprylic acid (C8:0)	ND
Crude fibre	6.58	Capric acid (C10:0)	0.33
Neutral detergent fibre	30.62	Undecanoic acid (C11:0)	ND
Nitrogen free extract	7.58	Lauric acid (C12:0)	9.17
Calcium	3.59	Tridecanoic acid (C13:0)	0.02
Phosphorus	0.70	Myristic acid (C14:0)	2.29
Chitin	4.32	Pentadecanoic acid (C15:0)	0.07
Essential amino acids		Palmitic acid (C16:0)	7.81
Isoleucine	1.61	Heptadecanoic acid (C17:0)	0.07
Leucine	2.61	Steric acid (C18:0)	1.33
Lysine	2.31	Unsaturated fatty acids (USFA)	
Methionine	0.58	Palmitoleic acid (C16:1n7)	1.08
Phenylalanine	1.51	cis-10-heptadecanoic acid (C17:1n10)	0.05
Threonine	1.47	cis-9-oleic acid (C18:1n9)	6.33
Tryptophan	0.42	cis-9,12-linoleic acid (C18:2n6)	3.65
Tyrosine	1.77	α-linolenic acid (C18:3n3)	0.28
Histidine	1.09	γ-linolenic acid (C18:3n6)	ND
Valine	2.44	cis-11,14-eicosadienoic acid (C20:2)	ND
Total	15.81	cis-11,14,17-eicosatrienoic acid (C20:3n3)	0.06
Amino acid recovery (%)^a^	44.06	Eicosapentaenoic acid (C20:5n3)	0.14
Non-essential amino acids		Erucic acid (C22:1n9)	ND
Alanine	3.24		
Arginine	1.45	Docosadienoic acid (C22:2)	ND
Aspartic acid	3.25	Docosahexaenoic acid (C22:6n3)	0.01
Cystine	ND	Nervonic acid (C24:1n9)	0.02
Glutamic acid	4.39	Total SFA	21.09
Glycine	2.08	Total USFA	11.62
Serine	1.71		
Proline	2.47		
Total	18.59		

*GE* gross energy, *SFA* saturated fatty acids, *USFA* unsaturated fatty acids, *ND* not detected

^a^Amino acid recovery = (total amino acids/% CP) × 100

### Animals, treatments, and management

A total of 80 piglets [(Landrace × Large White) × Duroc] weaned at 28 ± 3 days of age (7.34 ± 0.21 kg body weight, BW) were divided into four experimental groups and fed the following diets: PC, basal diet supplemented with 0.02% amoxicillin; NC, basal diet without supplementation; BSFL12, basal diet supplemented with 12% full-fat BSFL; and BSFL + Pro, basal diet supplemented with BSFL + 0.1% multi-probiotics. Each treatment had five replicates. Each animal pen had an equal number of gilts and barrows (1:1 ratio) in a randomised complete block design with initial body weight as the blocking factor. Pigs were housed in pens with slatted concrete flooring (20 pens, 1.6 × 2.1 m; stocking density, 0.8 m^2^/pig) furnished with a low-pressure nipple drinker, stainless steel trough, and heating lamp. Rice straw and gunny bags were supplied as bedding materials during the 14-day post-weaning period and were changed twice daily (0600 and 1900), following the guidelines of the EU Directive 2010/63/EC for animal experiments. All pigs were vaccinated against Aujeszky’s disease, salmonellosis, and transmissible gastroenteritis. Mash diets were formulated to meet or exceed the nutrient requirements for pigs weighing 11–25 kg. The diets were formulated in two phases (Phase I, 1–2 weeks post-weaning; Phase II, 3–4 weeks post-weaning) as suggested by the National Research Council (NRC) [15] (Table 2). All pigs had free access to feed and water throughout the experimental period.

**Table 2:** Ingredients and nutrient values of the experimental diet (% as fed basis)

Ingredient^a^	Phase I (day 1 to 14)				Phase II (day 15 to 28)			
	PC	NC	BSFL12	BSFL + Pro	PC	NC	BSFL12	BSFL + Pro
Corn	40.39	40.41	40.07	39.88	50.57	50.59	50.84	50.67
Soybean meal (43.8%)	28.88	28.88	17.22	17.31	31.39	31.39	19.80	19.87
Broken rice	10.00	10.00	10.00	10.00	6.00	6.00	6.00	6.00
Full-fat soybean meal	8.00	8.00	8.00	8.00	3.50	3.50	3.50	3.50
Whey powder	5.00	5.00	5.00	5.00	2.00	2.00	2.00	2.00
Skimmed milk	5.00	5.00	5.00	5.00	3.00	3.00	3.00	3.00
Black soldier fly larva	0.00	0.00	12.00	12.00	0.00	0.00	12.00	12.00
*Bacillus* probiotic^b^	0.00	0.00	0.00	0.10	0.00	0.00	0.00	0.10
Amoxicillin	0.02	0.00	0.00	0.00	0.02	0.00	0.00	0.00
L-Lysine HCl (78%)	0.29	0.29	0.29	0.29	0.34	0.34	0.34	0.34
DL-Methionine (99%)	0.09	0.09	0.09	0.09	0.11	0.11	0.11	0.11
Dicalcium phosphate	1.39	1.39	1.39	1.39	1.56	1.56	1.56	1.56
Limestone	0.09	0.09	0.09	0.09	0.66	0.66	0.00	0.00
Sodium chloride	0.35	0.35	0.35	0.35	0.35	0.35	0.35	0.35
Vitamin-mineral premix^c^	0.50	0.50	0.50	0.50	0.50	0.50	0.50	0.50
Total	100	100	100	100	100	100	100	100
Calculated values (%)								
ME (kcal/kg)^d^	3290.63	3291.30	3368.79	3335.39	3224.27	3224.93	3324.37	3320.98
Crude protein	22.50	22.50	22.50	22.50	21.50	21.50	21.50	21.50
Ca	0.68	0.68	0.68	0.68	0.80	0.80	0.80	0.80
Total phosphorus	0.66	0.66	0.66	0.66	0.68	0.68	0.68	0.68
Lysine	1.45	1.45	1.45	1.45	1.38	1.38	1.38	1.38
Methionine + Cysteine	0.65	0.65	0.65	0.65	0.65	0.65	0.65	0.65
Tryptophan	0.22	0.22	0.22	0.22	0.23	0.23	0.23	0.23
Threonine	0.87	0.87	0.87	0.87	0.86	0.84	0.84	0.88
Crude fat	3.83	3.83	7.53	7.53	3.30	3.72	7.02	7.02
Crude fibre	3.41	3.41	2.61	2.61	3.64	3.64	2.87	2.87
Chitin	0.00	0.00	0.52	0.52	0.00	0.00	0.52	0.52
Analysed values (%)								
GE (kcal/kg)	4363	4366	4424	4436	4354	4331	4452	4430
Crude protein	22.32	22.30	21.85	22.52	21.63	21.36	21.53	21.64
Ether extract	3.62	3.72	8.13	8.16	3.37	3.84	7.22	7.33

*GE* gross energy, *ME* metabolisable energy

^a^Calculated values were obtained from the nutrient composition data from actual analysis and the NRC [15]

^b^Probiotic mixture consisted of *Bacillus subtilis, B. licheniformis*, and *Saccharomyces cerevisiae*

^c^Provided (per kg of complete diet): vitamin A 8000 IU; vitamin D_3_ 1600 IU; vitamin E 34 IU; biotin 64 g; riboflavin 3.4 mg; calcium pantothenic acid 8 mg; niacin 16 mg; vitamin B_12_ 12 g; vitamin K 2.4 mg; Se as Na_2_SeO_3_ 0.1 mg; I as KI 0.32 mg; Mn as MnSO_4_ 25.2 mg; Cu as CuSO_4_ 53.9 mg; Fe as FeSO_4_ 127.3 mg; Zn as ZnSO_4_ 83.46 mg, and Co as CoSO_4_ 0.28 mg

^d^The ME value of BSFL was obtained from previous studies [16–18]

### Measurement of growth performance and incidence of diarrhoea

On days 1, 15, and 29 post-weaning, each pig was weighed individually at 0600. Following this, pen-based feed disappearance was recorded to determine the average daily gain (ADG), average daily feed intake (ADFI), and gain-to-feed ratio (G:F, calculated as ADG/ADFI). The severity of diarrhoea was measured visually using a faecal consistency score: 0, firm and shape faeces; 1, soft and shaped faeces; 2, loose faeces; and 3, watery faeces. Scores of 0 and 1 represented normal faeces, whereas scores of 2 and 3 represented diarrhoea. Diarrhoeal rate (%) was calculated using the following formula:

1 $$\textrm{Diarrhoeal}\ \textrm{rate}=\textrm{number}\ \textrm{of}\ \textrm{pigs}\ \textrm{with}\ \textrm{diarrhoea}/\left(\textrm{total}\ \textrm{number}\ \textrm{of}\ \textrm{pigs}\ \textrm{by}\ \textrm{treatment}\times \textrm{days}\ \textrm{with}\ \textrm{diarrhoea}\right)\times 100$$

### Apparent total tract digestibility (ATTD)

A total of 20 barrows (initial average BW = 10.26 ± 2.36 kg) were chosen separately from the feeding trial and assigned to each dietary treatment in five replicates in a completely randomised design. The pigs were housed individually in cages (0.58 m × 0.83 m) equipped with a grid and slurry pit for a 7-d adaption and 5-d faecal collection period. The experimental diets were offered at 12-h intervals in an amount equalling 3× the maintenance energy requirement (106 kcal of metabolisable energy per kg of BW^0.75^) [15]. During the faecal collection period, two indigestible indicators, chromic oxide and ferric oxide were homogenously mixed with all the experimental diets (5 g/kg feed of each marker) at the first and last meal, respectively, according to the marker-to-marker method [19]. The collection of faecal output started and ended when the initial and final marker appeared in the faeces, respectively. The faecal collection was performed daily at 1900 hrs. Pooled faeces were weighed and dried in a forced air-drying oven (60 °C for 72 h) and subsequently ground in a hammer mill using a 0.88 mm screen. Representative samples of pooled faeces and diets were used to measure the levels of dry matter (DM, method #930.15), CP (method #984.13), crude ash (method #942.15), and EE method (#920.39) using standard Association of Official Analytical Chemists protocols [20]. These values were used to calculate ATTD using following formula [19]:

2 $$\textrm{X}\ \textrm{apparent}\ \textrm{digestibility}=\frac{\textrm{X}\ \textrm{ingested}-\textrm{X}\ \textrm{excreted}}{\textrm{X}\ \textrm{ingested}}\times 100$$

where X represents DM, CP, crude ash, or EE in feed and faeces, respectively.

### Blood collection and analyses

On day 29, one healthy pig per pen (*n* = 20), weighing the BW average of the pen, was selected for blood sampling (10 mL) from the anterior vena cava. Blood samples were collected in serum-coated tubes with silica (Grener Bio-one, Chonburi, Thailand), allowed to clot at room temperature for 60 min, centrifuged for 10 min at 4 °C at 13,000×*g*, and then frozen at − 20 °C. Serum concentrations of immunoglobulin A (IgA), immunoglobulin G (IgG), immunoglobulin M (IgM), interleukin-1β (IL1β), interleukin-6 (IL6), and tumour necrosis factor alpha (TNFα) were quantified using porcine enzyme-linked immunosorbent kits (Abcam, Cambridge, UK). The concentrations of total antioxidant capacity (TAC), superoxide dismutase (SOD), glutathione peroxidase (GSH-Px), and malondialdehyde (MDA) were determined using commercial kits (Sigma-Aldrich, St. Louis, MO). All assays were performed as outlined by the manufacturer and conducted in triplicate to control for variation.

### Organ weight

After blood collection, all selected pigs were slaughtered after 12 h of fasting. The digestive tract was eviscerated to harvest the heart, liver, kidney, stomach, and spleen. Each organ was separately flushed with 0.9% phosphate-buffer saline solution, blot-dried, and weighed using a digital scale. Segments of the colon, cecum, and small intestine were also collected for later examination.

### Digesta pH

The cecum and colon (proximal, middle, and distal portions) tissues collected were immediately used to measure the hindgut pH with a pH metre (AP 110, Fisher Scientific, Pittsburgh, PA, USA).

### Intestinal histomorphology

Segments of the small intestine were collected immediately after euthanasia. Longitudinal dissections of the duodenum (50 cm caudal to pyloric sphincter), jejunum (5 cm from the pyloric sphincter), and ileum (20 cm from the ileocecal orifice) were performed carefully. Each sample was rinsed with saline solution and fixed with a neutral buffer (pH 7.0) and formaldehyde solution (10% vol/vol) for 72 hours. The tissue samples were embedded with ethanol and xylene and then transversely cut into 5-μm-thick sections using a rotary microtome (Leica RM2235, Wetzlar, Germany). The tissue sections were placed on glass slides and stained with haematoxylin-eosin (H&E staining, Sigma-Aldrich, St Louis, MO, USA). In total, 20 well-oriented villi (per stained section) and crypt columns were used to determine villus height (VH, from the tip of the villus to the basolateral membrane) and crypt depth (CD, from the villus–crypt junction and the submucosa) using an optical light microscope at 10× magnification. Average VH and CD values were recorded, and the villus height-to-crypt depth ratio (VH/CD) was calculated.

### Microbial count

Faecal samples were collected by rectal massage of the pigs (5 samples per treatment) and suspended in 0.9% (w/v) sodium chloride solution at 1:10 dilution. A 0.1 mL aliquot of each dilution was spread-plated in triplicate onto deMan, Rogosa, and Sharpe (Difco™ *Lactobacillus* MRS agar, Becton, Dickinson and Company, USA), MacConkey (Himedi™, HiMedia Laboratories, India), and *Salmonella*–*Shigella* agar (Oxoid™ SS Agar, Oxoid Limited, Thermo Fisher Scientific Inc., UK) for the determination of *Lactobacillus* spp., *Escherichia coli,* and *Salmonella* spp., respectively, following the manufacturer’s guidelines. The average growth of each microbe was log-transformed and represented as log_10_ colony-forming unit (CFU)/g of faeces.

### Statistical analysis

Data were analysed using general linear models in SAS (version 9.4, SAS Inst. Inc., Cary, NC, USA) using a randomised complete block design with “pen” as the experimental unit for growth performance and diarrhoea rate, and “each pig” as the experimental unit for digestibility, blood analyses, organ weight, hindgut pH, intestinal morphology, and microbial counts. The statistical model was:

3 $$Yij=\mu +{\alpha}_i+{\beta}_j+{\varepsilon}_{ij}$$

where Y*ij* is the *j*th observation of the *i*th treatment; *μ* is the overall mean; *α*_*i*_ is the effect of the *i*th block (i = 1–5); *β*_*j*_ is the effect of the *j*th treatment (*j* = 1–4); and ε_ij_ is the error term.

Normal probability was assessed using the Shapiro-Wilk test and homogeneity of error variance among treatments was verified using the Hartley’s test. If the data was not characterised by the normal distribution, the nonparametric analysis was verified using the Friedman’s test. Duncan’s new multiple range test was used to test for significant differences among dietary treatments using the SAS package. All data were represented as the mean ± standard error of the mean (SEM), and *P* < 0.05 was considered statistically significant.

## Results

### Growth performance and diarrhoeal rate

During the experimental period, the values of ADG (*P* = 0.013) and G:F (*P* = 0.032) were increased by 18.16 and 18.21%, respectively, compared to those obtained using the NC diet (Table 3). Outcomes of the BSFL + Pro diet were comparable to those of the positive control diet (PC, basal diet supplemented with 0.02% amoxicillin) in all experimental periods. However, the BW, ADFI and the incidence of diarrhoea was unaffected by dietary treatment during Phases I and II (*P* > 0.05).

**Table 3:** Effect of dietary BSFL full-fat meal and probiotic mixture to replace antibiotics on growth performance and diarrhoeal rate in weaning pigs^1,2^

	Item	Dietary treatment				Normality	Homogeneity of error variance		
		PC	NC	BSFL12	BSFL+ Pro	*P* - value	*P* - value	SEM	*P* - value
D0–14	BW d0 (kg)	7.32	7.36	7.35	7.34	0.839	0.025	0.208	0.782
	BW d15 (kg)	10.46	9.97	10.32	10.78	0.090	0.038	0.236	0.095
	ADG (g)	224.68	186.57	212.28	245.86	0.380	0.121	13.563	0.056
	ADFI (g)	389	392	387	403	0.216	0.109	11.742	0.788
	G:F	0.581	0.476	0.550	0.613	0.273	0.310	0.039	0.137
D15–28	BW d29 (kg)	17.43	16.06	16.92	17.87	0.784	0.010	0.297	0.077
	ADG (g)	497.68	434.78	471.96	506.36	0.421	0.114	26.512	0.272
	ADFI (g)	694	676	681	670	0.015	0.022	7.676	0.156
	G:F	0.718	0.644	0.692	0.736	0.733	0.099	0.041	0.443
d0–28	ADG (g)	361.18^a^	310.68^b^	341.82^ab^	376.11^a^	0.592	0.087	11.996	0.013
	ADFI (g)	541	534	534	546	0.430	0.004	5.604	0.374
	G:F	0.668^a^	0.582^b^	0.641^ab^	0.688^a^	0.620	0.175	0.023	0.032
	Diarrhoea rate (%)^3^	5.42	12.58	6.83	4.28	0.031	0.031	1.968	0.323

^1^*Abbreviations*: *PC* basal diet with amoxicillin 0.02%, *NC* basal diet without addition, *BSFL12* basal diet plus 12% black solder fly larva full-fat meal, *BSFL* + *Pro* basal diet plus 12% black solder fly larva full-fat meal and 0.1% multi-probiotics, *BW* body weight, *ADG* average daily gain, *ADFI* average daily feed intake, and *G:F* gain-to-feed ratio

^2^Values shows means of five replicates (pen) per treatment

^3^Data present the average value from days 1 to 28 ^a,b^Values within a row not sharing common lowercase superscripts differ significantly (*P* < 0.05)

### Nutrient digestibility coefficients

Diets supplemented with BSFL12 (basal diet supplemented with 12% BSFL full-fat meal) and BSFL + Pro groups increased the ATTD of DM (*P* = 0.047), CP (*P* = 0.022), and EE (*P* = 0.019) compared to that seen in the NC-fed group (Table 4); however, ATTD was comparable to that in the PC-fed group. However, there were no differences in the ATTD of crude ash among dietary treatments (*P* > 0.05).

**Table 4:** Effect of dietary BSFL full-fat meal and probiotic mixture to replace antibiotics on the apparent total tract digestibility (ATTD) in weaning pigs on day 28^1,2^

Item	Dietary treatment				Normality	Homogeneity of error variance		
	PC	*P* - value	BSFL12	BSFL+ Pro	*P* - value	*P* - value	SEM	*P - value*
Dry matter	88.20^ab^	83.65^b^	90.82^a^	90.32^a^	0.078	0.220	1.642	0.047
Crude protein	83.57^ab^	77.39^b^	87.51^a^	86.72^a^	0.274	0.131	1.995	0.022
Crude ash	53.06	52.56	61.52	62.97	0.010	0.053	7.865	0.753
Ether extract	67.89^ab^	57.64^b^	79.32^a^	75.06^a^	0.174	0.189	3.987	0.019

^1^*Abbreviations*: *PC* basal diet with amoxicillin 0.02%, *NC* basal diet without addition, *BSFL12* basal diet plus 12% black solder fly larva full-fat meal, *BSFL* + *Pro* basal diet plus 12% black solder fly larva full-fat meal and 0.1% multi-probiotics

^2^Values shows means of five replicates (pen) per treatment

^a,b^Values within a row not sharing common lowercase superscripts differ significantly (*P* < 0.05)

### Blood-related gut health and antioxidative stress

The concentration of IgA (*P* = 0.044) was higher in the BSFL-supplemented diets, whereas reduced IL1β (*P* = 0.031) and MDA (*P* = 0.006) concentrations occurred in comparison to NC (Table 5). In addition, the pigs fed BSFL+ Pro diet had higher IgG (*P* = 0.008), SOD (*P* = 0.033), and GSH-Px (*P* = 0.026), with a lower secretion of TNFα than those fed the NC diet (*P* = 0.044). However, the dietary treatments did not affect serum concentrations of IgM, IL6, and **TAC** (*P* > 0.05).

**Table 5:** Effect of dietary BSFL full-fat meal and probiotic mixture to replace antibiotic on the blood-related gut health and antioxidative stress in weaning pigs^1,2^

Item	Dietary treatment				Normality	Homogeneity of error variance		
	PC	NC	BSFL12	BSFL+ Pro	*P* - value	*P*- value	SEM	*P* - value
IgA (g/L)	0.77^ab^	0.52^b^	0.84^a^	0.93^a^	0.195	0.282	0.092	0.044
IgG (g/L)	17.68^b^	12.84^b^	21.02^b^	25.79^a^	0.011	0.001	2.013	0.008
IgM (g/L)	2.15	1.97	2.09	2.13	0.112	0.037	0.143	0.896
IL1β (pg/mL)	298.20^ab^	343.97^a^	274.96^b^	243.13^b^	0.612	0.163	20.777	0.031
IL6 (pg/mL)	132.79	144.67	123.15	116.21	0.126	0.071	10.687	0.309
TNFα (pg/mL)	52.84^ab^	74.83^a^	54.54^ab^	39.13^b^	0.146	0.043	7.695	0.106
TAC (U/mL)	10.32	8.48	13.64	12.86	0.571	0.138	2.431	0.447
SOD (U/mL)	68.13^b^	51.46^b^	78.32^ab^	89.38^a^	0.157	0.012	8.775	0.033
GSH-Px (U/mL)	650.36^b^	552.65^b^	878.98^ab^	886.66^a^	0.024	0.142	78.977	0.026
MDA (nmol/mL)	7.51^ab^	8.84^a^	6.31^bc^	5.39^c^	0.654	0.099	0.568	0.006

^1^*Abbreviations*: *PC* basal diet with amoxicillin 0.02%, *NC* basal diet without addition, *BSFL12* basal diet plus 12% black solder fly larva full-fat meal, *BSFL* + *Pro* basal diet plus 12% black solder fly larva full-fat meal and 0.1% multi-probiotics, *IgA* immunoglobulin A, *IgG* immunoglobulin G, *IgM* immunoglobulin M, *IL1β* interleukine-1β, *IL6* interleukine-6, *TNFα* tumour necrosis factor alpha, *TAC* total antioxidant capacity, *SOD* superoxide dismutase, *GSH-Px* glutathione peroxidase, *MDA* malondialdehyde

^2^Data are shown as group mean ± standard error of mean (SEM) (*n* = 5 per treatment)

^a-c^Values within a row not sharing common lowercase superscripts differ significantly (*P* < 0.05)

### Organ weight

Pigs that were fed the BSFL + Pro diet had the highest spleen weight compared to pigs who were fed other treatments (*P* = 0.011; Table 6). However, the organ weights of heart, liver, kidney, and stomach were unaffected by dietary treatment (*P* > 0.05).

**Table 6:** Effect of dietary BSFL full-fat meal and probiotic mixture to replace antibiotics on organ weight in weaning pigs^1,2^

Item	Dietary treatment				Normality	Homogeneity of error variance		
	PC	NC	BSFL12	BSFL+ Pro	*P* - value	*P*-value	SEM	*P - value*
Heart	4.07	4.21	4.19	4.42	0.330	0.007	0.452	0.724
Liver	27.48	33.08	30.27	28.33	0.406	0.036	3.961	0.896
Kidney	3.99	4.59	4.23	3.71	0.568	0.177	0.335	0.338
Stomach	8.51	8.38	7.79	8.94	0.013	0.004	0.784	0.564
Spleen	2.09^b^	2.08^b^	2.49^b^	3.12^a^	0.182	0.066	0.202	0.011

^1^*Abbreviations*: *PC* basal diet with amoxicillin 0.02%, *NC* basal diet without addition, *BSFL12* basal diet plus 12% black solder fly larva full-fat meal, *BSFL* + *Pro* basal diet plus 12% black solder fly larva full-fat meal and 0.1% multi-probiotics

^2^Data are shown as group mean ± standard error of mean (SEM) (n = 5 per treatment)

^a,b^Values within a row not sharing common lowercase superscripts differ significantly (*P* < 0.05)

### Digesta pH

The digesta pH in the caecal, proximal, middle, and distal small intestinal segments was not affected by any of the dietary treatments (*P* > 0.05; Table 7). However, a tendency for a lower pH value in the caecum was observed in the BSFL + Pro (*P* = 0.069) treatment.

**Table 7:** Effect of dietary BSFL full-fat meal and probiotic mixture to replace antibiotic on digesta pH in weaning pigs^a,b^

Item	Dietary treatment				Normality	Homogeneity of error variance		
	PC	NC	BSFL12	BSFL+ Pro	*P* - value	*P*-value	SEM	*P* - value
Cecum	4.51	6.29	5.08	4.23	0.018	0.086	0.420	0.069
Proximal colon	5.87	5.26	5.48	5.31	0.627	0.095	0.344	0.605
Middle colon	5.39	4.97	5.44	5.32	0.018	0.001	0.454	0.854
Distal colon	4.87	5.62	4.76	4.27	0.151	0.001	0.458	0.516

^a^*Abbreviations*: *PC* basal diet with amoxicillin 0.02%, *NC* basal diet without addition, *BSFL12* basal diet plus 12% black solder fly larva full-fat meal, *BSFL* + *Pro* basal diet plus 12% black solder fly larva full-fat meal and 0.1% multi-probiotics

^b^Data are shown as group mean ± standard error of mean (SEM) (n = 5 per treatment)

### Intestinal histomorphology

The VH and VH/CD in the duodenum were higher in the BSFL-supplemented treatments *(P* = 0.033, and 0.004, respectively; Table 8). However, the duodenum CD and all intestinal histomorphology measurements in the jejunum and ileum were similar among all treatments (*P* > 0.05).

**Table 8:** Effect of dietary BSFL full-fat meal and probiotic mixture to replace antibiotics on the intestinal histomorphology in weaning pigs^1,2^

	Item	Dietary treatment				Normality	Homogeneity of error variance		
		PC	NC	BSFL12	BSFL + Pro	*P* - value	*P*-value	SEM	*P - value*
Duodenum	Villus height (μm)	701.96^ab^	578.18^b^	770.45^a^	774.98^a^	0.725	0.163	45.527	0.033
	Crypt depth (μm)	366.39	447.73	339.18	358.72	0.003	0.035	21.277	0.077
	VH/CD	1.97^a^	1.34^b^	2.28^a^	2.16^a^	0.444	0.094	0.151	0.004
Jejunum	Villus height (μm)	671.86	596.11	663.87	707.31	0.034	0.264	76.534	0.668
	Crypt depth (μm)	370.53	339.16	347.31	357.41	0.113	0.351	26.434	0.851
	VH/CD	1.94	1.85	1.95	2.29	0.117	0.014	0.317	0.896
Ileum	Villus height (μm)	487.78	478.07	484.32	534.34	0.375	0.184	38.289	0.719
	Crypt depth (μm)	297.21	271.84	262.69	298.61	0.021	0.114	43.468	0.564
	VH/CD	1.76	1.87	2.06	2.11	0.096	0.141	0.368	0.898

^1^*Abbreviations*: *PC* basal diet with amoxicillin 0.02%, *NC* basal diet without addition, *BSFL12* basal diet plus 12% black solder fly larva full-fat meal, *BSFL + Pro* basal diet plus 12% black solder fly larva full-fat meal and 0.1% multi-probiotics, *VH/CD* villus height-to-crypt depth ratio

^2^Data are shown as group mean ± standard error of mean (SEM) (n = 5 per treatment)

^a,b^Values within a row not sharing common lowercase superscripts differ significantly (*P* < 0.05)

### Microbial count

The BSFL + Pro treatment increased faecal *Lactobacillus* spp. Count by 29.26 and 64.90% compared with that seen with the PC and NC treatments, respectively (*P* = 0.008; Table 9). Simultaneously, faecal *E. coli* count decreased in the BSFL + Pro treatment group in comparison to that in the NC group (*P* = 0.021). No differences were detected in the faecal *Salmonella* spp. counts among the dietary treatments (*P* > 0.05).

**Table 9:** Effect of dietary BSFL full-fat meal and probiotic mixture to replace antibiotics on the microbial count (log colony-forming unit [CFU]/g faeces) in weaning pigs^1,2^

Item	Black soldier fly larva (%)				Normality	Homogeneity of error variance		
	PC	NC	BSFL12	BSFL+ Pro	*P* - value	*P*-value	SEM	*P* - value
*Lactobacillus* spp.	7.45^bc^	5.84^c^	8.71^ab^	9.63^a^	0.316	0.071	0.652	0.008
*Escherichia coli*	6.08^b^	8.61^a^	6.92^ab^	5.72^b^	0.585	0.093	0.592	0.021
*Salmonella* spp.	5.58	7.23	6.51	5.97	0.476	0.095	0.564	0.239

^1^*Abbreviations*: *PC* basal diet with amoxicillin 0.02%, *NC* basal diet without addition, *BSFL12* basal diet plus 12% black solder fly larva full-fat meal, *BSFL + Pro* basal diet plus 12% black solder fly larva full-fat meal and 0.1% multi-probiotics

^2^Data are shown as group mean ± standard error of mean (SEM) (*n* = 5 per treatment)

^a-c^Values within a row not sharing common lowercase superscripts differ significantly (*P* < 0.05)

## Discussion

### Growth performance and diarrhoeal rate

The extensive in-feed administration of antibiotics in swine production and lack of awareness regarding its effects are driving the accelerated and widespread emergence of antibiotic-resistant bacteria [11]. Dietary supplements, BSFL full-fat meal, and multi-probiotics have been proposed as substitutes for antibiotics for future generations of weanling pigs. However, the combined effects of these supplements on overall growth performance and the incidence of diarrhoea in piglets have not well-established. In this study, these two combinations demonstrated improvements in growth performance and feed efficiency. Although the underlying mechanism remains unknown, it may have exerted an effect because of the unique properties of the diet in not only providing an excellent source of protein, essential amino acids, and energy but also providing several active compounds [2, 6, 7]. Supplementation positively enhanced the overall growth performance and health benefits for the weaned pigs. According to Jin et al. [21], dietary supplementation with 8% BSFL full-fat meal improves overall growth performance in a dose-dependent manner. However, BSFL-supplemented diets did not affect the growth performance of piglets when fed at a concentration below 4% [22]. The BSFL full-fat meal is normally relatively high in lauric acid (12.83% DM) [5], which accounted for ~ 1.52% of our calculated value in the BSFL-supplemented diet. The chitin content will trigger the secretion of growth hormone and insulin-like growth factor 1, leading to a heavier body weight in weaning pigs [23]. Other factors might be associated with the action of AMPs in promoting ADG when supplied at of 2 mg/kg AMPs [24]. However, there is no available data of insect-derived AMPs in improving growth performance and feed efficiency in monogastrics; therefore, further research is needed to elucidate the in vivo effects. Taken together with probiotic mixtures, strong antimicrobial and growth-promoting effects may be expected at inclusions of > 0.1% [13].

In the present study, we found that, compared with the NC diet, supplementation with BSFL alone did not exert considerable effect on the growth performance of pigs in any period. However, the combination of BSFL full-fat meal can be used to decrease the quantity of soybean meal (28.88% vs. 17.31%) in diets for weaned piglets. This will have the added value of essential amino acid recovery over soybean meal (44.06% vs. 41.30%) [15], leading to better protein utilisation with less undigestible protein loss. Furthermore, the higher EE in Phase I (3.83% vs. 7.53%) and II (3.72% vs. 7.02%) in the BSFL-supplemented diets may positively increase nutrient uptake.

Addition of the *Bacillus* spp. probiotic mixture has been proposed to produce several active enzymes in the digestion of soluble or insoluble fractions of feed that will rapidly increase the absorbability of nutrients by enterocytes and their ability to maintain health status of pigs during the post-weaning period [25]. These unique characteristics facilitate faster gut recovery and better nutrient uptake to support growth performance of the weaning pigs. This was evidenced by the increase in BW and ADG in the BSFL+ Pro-supplemented diet.

### ATTD

The inclusion of insect protein (BSFL) and multi-probiotics has been shown to increase the nutrient digestibility of DM, CP, and EE in weaned pigs [26]. Previous studies have also reported that *B. licheniformis* and *B. subtilis* produce several extracellular enzymes that are beneficial to hosts, including α-amylase, proteinase, lipase, xylanase, cellulase, and pectinase [27]. Indeed, *B. subtilis* exceeds *B. licheniformis* in producing glycosyl hydrolase, which assists in the degradation of glycosidic linkages in complex sugars [28]. BSFL full-fat meal has a high protein content and is a good source of amino acids, which can further improve CP digestibility [29]. Inclusion of BSFL at 12% had no effect on protein digestibility, indicating the possibility of replacing soybean meal with BSFL full-fat meal as a protein source. This increases the digestion and absorption of nutrients in the dietary treatment supplemented with BSFL full-fat meal.

The high fat content and degree of saturation of fatty acids may be a major reason for the improved digestibility of EE in BSFL-supplemented diets over in NC diets (7.02–7.53% vs. 3.72–3.83%). In this study, C12:0—which is rapidly utilised and passively absorbed by the animals—was the main component of saturated fatty acids in the BSFL12 diet. Combined with microbial lipase secretion from *Bacillus*-based probiotics, C12:0 may have broad effects in the hydrolysed 1- and 3- positions of dietary triglycerides. Although the experimental dietary combinations showed improved nutrient digestibility compared to the NC diet, their effects were comparable to those of the PC diet, suggesting the greater availability of nutrients beyond the antibiotic treatment.

### Blood-related gut health and antioxidative stress

Serum immunoglobulins can be used to determine cellular responses and the ability of an animal’s body to recognise pathogenic invasion [30]. Our study showed that serum IgA concentrations increased in pigs fed the BSFL12 and BSFL + Pro diets. Serum IgA can subsequently interact with lactoferrin and/or transferrin for bacteriostatic action by increasing the adhesion to epithelial cells (to improve their adhesion to the mucus) and neutralising bacterial toxins. Therefore, increased levels of serum IgA prompt the initial defence against infection by pathogens [31]. Serum IgG inhibits various stressors, including diseases and intestinal disorders, during the first week post-weaning [32]. Therefore, higher levels of serum IgG can suppress invading pathogens and activate long-lasting immunity. The feedback regulation of IgG also induces the secretion of novel IgG antibodies via the activation of B lymphocytes [31]. This is consistent with previous reports that dietary inclusion of *B. subtilis* will increase serum IgA and IgG levels in rabbits and neonatal piglets [33, 34]. The higher C12:0 content of BSFL-supplemented diets may also activate interleukin production, which in turn promotes the production of immunoglobulins [35]. However, the mechanism by which BSFL (alone or in combination with *Bacillus*-based probiotics) promotes immunoglobulin secretion is still unclear. It is possible that the binding site of *Bacillus*-based probiotics can bind with IgG Fc-receptors as additional ligands, which can subsequently influence immunoglobulin secretion [36].

Pro-inflammatory cytokines (such as IL1β, IL6, and TNFα) are known to be secreted as part of the innate immune response [37]. These factors have detrimental effects on intestinal mucosal injury and dysfunction, impair nutrient digestion and absorption, and subsequently lead to a poor growth rate [37]. However, dietary BSFL can activate intestinal development, immunity, and anti-inflammation [37, 38] via the direct utilisation of C12:0 by enterocytes for energy production, thereby maintaining the integrity of the intestinal mucosa in young piglets [39]. Peptidoglycans in the insect skeleton can also attach to the binding sites of pathogens, thus triggering IgA release into the intestinal lumen and inhibiting the secretion of pro-inflammatory factors. Chitin and its derivatives have also been reported to polarise faecal calprotectin, which is a sensitive and non-invasive marker of active inflammation in the gastrointestinal tract. They also regulate a main receptor for commensal recognition in gut innate immunity [40], thus reducing inflammation in the lower intestine. It is unclear how BSFL supplementation lowers the serum TNFα concentration. Although possible factors may be attributed to the molecular weight, degree of saturation, particle size, source, and the purification level of the BSFL [41], further research is warranted. Our study observed a positive response on lowering TNFα when applied with the *Bacillus*-based probiotic combination. It is well-established that *Bacillus* genera can synthesise bacteriocins, which have a widely antimicrobial action, to suppress the growth of several gram-positive bacteria via the permeability of their cytoplasmic membrane [42], thereby decreasing the penetration of the inflammatory cells. Furthermore, *B. subtilis* has a stronger ability to enhance innate immunity via β-defensin, which can have a direct effect on pathogenic colonisation and more predominantly involve the initiation and regulation of the adaptive immune response [34]. We thus propose that the decreased pro-inflammatory and increased anti-inflammatory cytokines caused by the *Bacillus-*based probiotic mixture may be linked to the capacity of these bacteria to decrease the increased inflammatory status associated with the transition at weaning.

An increase in the antioxidant enzyme activity of SOD and GSH-Px is also associated with antioxidant defence mechanisms. The SOD is an effective antioxidant enzyme for radical detoxification at the beginning of the free radical formation, whereas the enzymatic GSH-Px effectively inhibits the harmful accumulation of intracellular hydrogen peroxide, thus preventing damage to DNA, proteins, and membrane lipids in the animal body. The BSFL has appropriate amount of total phenolic compounds (32–35 mg/gallic acid equivalents g DM), whereas action of the *Bacillus* probiotic mixture (caused by the utilisation of antioxidant enzymes), modulates the host’s antioxidant system and probiotic-mediated antioxidant signalling mechanism [43]. This contributes to the scavenging of reactive oxygen species [44]. This is consistent with the increase in antioxidant enzymes and decrease in MDA concentrations, as shown in the current study. Therefore, it is suggested that the active substances derived from BSFL and probiotic mixtures may have been absorbed sufficiently for activating antioxidant enzymes in experimental pigs.

### Organ weight

The spleen is an important immune organ that can be used to evaluate immune responses in weaned pigs. Changes in the immune organ index indicate immune function and resistance to pathogenic invasion in animals. In the present study, the relative weight of the spleen increased after 4 weeks of feeding with BSFL + Pro. Previous studies have reported an increase in the spleen weight following the dietary inclusion of *B. subtilis* (1 × 10^6^ cfu/g) or chitin and its derivatives [34, 45]. This increase in spleen weight may enhance the immune function of weaned pigs, help them overcome weaning stress, and therefore improve their overall growth performance.

### Digesta pH

The digesta pH was substantially lower in the BSFL + Pro treatment group, indicating that these pigs were capable of generating acidic conditions to facilitate microbial growth and colonisation. The underlying mechanism of this process may be related to the large amount of chitin and its derivatives (rather than fibre content) passing through the large intestine [46], which causes a shift in hindgut fermentation and leads to the generation of short-chain fatty acids that supply energy for the growth of lactic acid-producing bacteria, thus lowering the number of pathogens [47]. This is consistent with our observation of faecal microbial counts in this study. However, the content of short-chain fatty acids should be assessed to explore this positive effect in more detail.

### Intestinal histomorphology

Changes in intestinal histomorphology—including VH, CD, and VH:CD—are indicative of gut health in pigs. A longer VH increases the mucosal surface area, facilitating improved digestion and absorption of available nutrients. Moreover, a shorter CD suppresses the rapid turnover of the intestinal epithelium, thus facilitating the renewal of villi in response to normal sloughing or inflammation from pathogenic invasion. The VH:CD ratio is typically associated with increased epithelial turnover [48]. Our results indicate that dietary BSFL12 and BSFL + Pro increased duodenal VH in weaned pigs. This may be because BSFL-supplemented diets promoted the activity of digestive enzymes (including membrane-bound peptidases) and facilitated more efficient utilisation of CP, amino acids, and EE [49]. This likely increased the availability of nutrients for the maturation of undifferentiated cells. Han et al. [38] also reported that chitin derivatives enhanced enterocyte proliferation and diminished villous atrophy. This suggests that the presence of 0.52% chitin in the BSFL full-fat meal diets can be efficiently utilised by chitin-degrading enzymes in the piglets’ stomach [50] and via hindgut microbial fermentation (47). Furthermore, the increased VH in the duodenum of pigs fed the BSFL full-fat meal diet suggests an increased surface area for greater digestion and absorption of the available nutrients. In addition, the combination with *Bacillus*-based probiotics has been reported to enhance various exogenous enzyme secretions including protease, lipase, phytase, and cellulase [51]. This action possibly increases the digestion and nutrient absorption of fat and CP from the BSFL full-fat meal, thus enhancing nutrient availability for greater differentiation of epithelial cells.

### Microbial count

The presence of calculated C12:0 in the BSFL-supplemented diet (~ 1.10%) is beneficial for the intestinal microbiota of weaning pigs, facilitates the semi-permeable membrane of pathogenic microbes, and improves the intestinal structure of piglets [52]. Therefore, in combination with chitin and its derivatives, C12:0 may have desirable effects on the gut microbiota of pigs. Chitin is a major fibrous compound in arthropod exoskeletons. It is strongly attached with β-glucan in the chitin–glucan complex, which is selectively used as a fibrous substrate by host microorganisms for their own growth [53]. This promotes the adherence of beneficial microbiota (such as *Lactobacillus* spp.) while reducing the faecal *E. coli* count in the hindgut [49, 54]. This is consistent with previous findings in which the inclusion of lower amounts of chitin had a greater influence on colonic microbiota than chitin supplementation in high amounts [55]. In addition, Yu et al. [56], demonstrated that pigs fed 4% BSFL showed a substantially higher abundance of *Lactobacillus* spp. than those fed 8% BSFL (0.19% vs. 0.37% chitin, respectively). This may indicate that feeding low levels of BSFL full-fat meal has desirable effects on the faecal microbiota of weaning pigs, compared to those on the PC diet. Interestingly, the combination of BSFL+ Pro produced a greater colonisation of *Lactobacillus* spp. and lower *E. coli* than both the control and NC diets, respectively. A previous study demonstrated that the addition of 0.1% multi-probiotics (consisting of *B. subtilis*, *B. licheniformis*, and *S. cerevisiae*) had a beneficial effect on maintaining faecal microbiota count by promoting growth of *Lactobacillus* spp. in growing pigs [57]. One possible explanation for this outcome is that *Bacillus*-based probiotics are considered facultative anaerobes with high resistance to acidic conditions (such as the gastrointestinal tract of animals) [58]. This unique characteristic may allow these microbes to attach with the gut surface and produce various bacteriolytic proteins [27]. This explanation is supported by our finding of the digesta pH being appropriate for the increased activity and proliferation of lactic acid-producing bacteria in this study. Another explanation is that it might be because of the interaction between the insect AMPs and microbial bacteriocins, and gut microbiota community. With the production of bacteriocins, *Bacillus* spp. probiotics have an inhibitory effect on pathogenic growth via the activation of their virulent genes and production of elastase and endopeptidase [59]. This mechanism can destroy the early cells of Gram-negative bacteria [59]. This is a potential inhibitor of *E. coli*, acting directly on the cell wall by inhibiting peptidoglycan or lipopolysaccharide O-antigen synthesis by permeabilising the membrane, resulting in ion leakage of the cellular content and cell death [60]. Taken together with insect AMPs, which include both hydrophobic and hydrophilic structures with a highly positive charge (ranging from + 2 to + 9) [61], they can interact with the negative charge of bacterial cell membrane. There is a concomitant increase in the permeability of the bacterial membrane, including interference with bacterial metabolism, disruption of bacterial cell integrity, and targeting of cytoplasmic components [61, 62], which ultimately retards gram-negative bacterial growth. It has been reported that *H. illucens* is an enriching source of AMPs, owing to the larval instar that feeds on decaying organic substrates and protects itself from the pathogens and restores substrate health via the reduction of *E. coil* [63]. It is suggested that the combinations of both substrates effectively enhanced the microbial populations in weaned pigs.

## Conclusion

The BSFL + Pro has the potential to activate IgG, GSH-Px, and *Lactobacillus* spp. growth more than the in-feed antibiotic treatment. Growth performance, nutrient digestibility coefficients, and intestinal histomorphology were comparable among the two treatments. Therefore, it is a viable alternative to antibiotics in nursery diets, without any impairment of growth performance and gut health in weaning pigs.

## Data Availability

All datasets are available from the corresponding author on reasonable request.

## Abbreviations

ADFI: Average daily feed intake
ADG: Average daily gain
ATTD: Apparent total tract digestibility
BSFL: Full-fat black soldier fly larvae
BSFL12: Negative control plus 12% BSFL full-fat meal
BSFL + Pro: BSFL full-fat meal plus 0.1% multi-probiotics
CD: Crypt depth
CP: Crude protein
DM: Dry matter
EE: Ether extract
GE: Gross energy
G:F: Gain-to-feed ratio
GSH-Px: Glutathione peroxidase
IgA: Immunoglobulin A
IgG: Immunoglobulin G
IgM: Immunoglobulin M
IL1β: Interleukin-1β
IL6: Interleukin-6
Kp: Nitrogen-to-protein conversion factor
MDA: Malondialdehyde
ME: Metabolisable energy
NC: Negative control
ND: Not detected
PC: Positive control
SFA: Saturated fatty acids
SOD: Superoxide dismutase
TAC: Total antioxidant capacity
TNFα: Tumour necrosis factor alpha
USFA: Unsaturated fatty acids
VH: Villus height
VH/CD: Villus height-to-crypt depth ratio
